# Differences in working memory coding of biological motion attributed to oneself and others

**DOI:** 10.1002/hbm.25879

**Published:** 2022-04-25

**Authors:** Mateusz Woźniak, Timo Torsten Schmidt, Yuan‐hao Wu, Felix Blankenburg, Jakob Hohwy

**Affiliations:** ^1^ Cognition and Philosophy Lab, Department of Philosophy Monash University Melbourne Australia; ^2^ Social Mind and Body Research Group, Department of Cognitive Science Central European University Vienna Austria; ^3^ Neurocomputation and Neuroimaging Unit (NNU), Department of Education and Psychology Freie Universität Berlin Berlin Germany; ^4^ Monash Centre for Consciousness & Contemplative Studies Monash University Melbourne Australia

**Keywords:** biological motion, body representation, mirror neurons, MVPA, self‐representation, self‐other, working memory

## Abstract

The question how the brain distinguishes between information about self and others is of fundamental interest to both philosophy and neuroscience. In this functional magnetic resonance imaging (fMRI) study, we sought to distinguish the neural substrates of representing a full‐body movement as one's movement and as someone else's movement. Participants performed a delayed match‐to‐sample working memory task where a retained full‐body movement (displayed using point‐light walkers) was arbitrarily labeled as one's own movement or as performed by someone else. By using arbitrary associations we aimed to address a limitation of previous studies, namely that our own movements are more familiar to us than movements of other people. A searchlight multivariate decoding analysis was used to test where information about types of movement and about self‐association was coded. Movement specific activation patterns were found in a network of regions also involved in perceptual processing of movement stimuli, however not in early sensory regions. Information about whether a memorized movement was associated with the self or with another person was found to be coded by activity in the left middle frontal gyrus (MFG), left inferior frontal gyrus (IFG), bilateral supplementary motor area, and (at reduced threshold) in the left temporoparietal junction (TPJ). These areas are frequently reported as involved in action understanding (IFG, MFG) and domain‐general self/other distinction (TPJ). Finally, in univariate analysis we found that selecting a self‐associated movement for retention was related to increased activity in the ventral medial prefrontal cortex.

## Graphical abstract

We conducted a functional magnetic resonance imaging (fMRI) study in which participants had to maintain self‐ and other‐attributed movements in working memory. MVPA analysis revealed that brain areas responsible for action understanding (IFG, MFG) and self/other distinction (TPJ) differentially coded self‐ than other‐attributed movements retained in working memory. Univariate analysis showed that selecting a self‐attributed movement for retention in working memory led to increased activation in vmPFC, an area responsible for processing of conceptual self‐related information. Our results help understand neural mechanisms underpinning bodily and conceptual self‐representations.

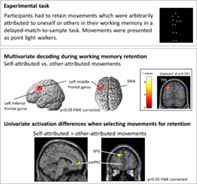

## 1 INTRODUCTION

The body is considered as the basis of our self—as Baumeister (1999) influentially remarked: “Everywhere in the world, self starts with body.” While the abstract, language‐mediated aspect of our self‐emerge only later in our lives, knowledge about our bodies is present from the onset of our existence. These two types of information have been argued to form two distinct self‐representations: the bodily self and the conceptual self (Christoff, Cosmelli, Legrand, & Thompson, 2011; Gallagher, 2000; Neisser, 1988). This difference also finds support in research on the brain mechanisms underpinning processing of bodily and abstract self‐related information. The bodily self is underpinned primarily by multisensory and sensorimotor brain systems, as shown by studies on rubber hand illusion, full body illusion, and recognition of one's face, body, and body movements (Blanke, 2012; Blanke, Slater, & Serino, 2015; Christoff et al., 2011; Limanowski & Blankenburg, 2013; Tsakiris, 2010, 2017). The conceptual self is mostly related to the activity of higher‐level brain areas, especially the cortical midline structures and has been investigated through evaluation of self‐related adjectives or formation of abstract, arbitrary self‐associations (Hu et al., 2016; Martinelli, Sperduti, & Piolino, 2013; Northoff et al., 2006; Qin & Northoff, 2011; Yin, Bi, Chen, & Egner, 2021). However, there is relatively little overlap between the brain areas involved in representing these two types of self‐related information (Hu et al., 2016; Qin, Wang, & Northoff, 2020).

Neurocognitive processing of information about one's bodily self is typically investigated through the use of images or videos of participants' face, body, body parts, or body movements. However, all of these stimuli are characterized not only by their self‐relatedness, but also by the fact that they are highly familiar to participants. Most previous studies have attempted to minimize the influence of familiarity by comparing processing of self‐related stimuli (own face, own movement pattern) with stimuli representing familiar or famous others (friends, family, celebrities). However, this kind of control conditions can never fully match with regard to familiarity. We spend all of our lives inside our bodies, while we devote substantially smaller fraction of lifetime paying attention to the bodies of other people, even famous ones. Therefore, while studies comparing processing of self‐related information with information related to familiar others provide an important contribution to our understanding of how the brain represents our bodily self, there is a need for validation of these results using designs which to a greater extent control for familiarity.

To control for familiarity in self‐related processing, recent studies have begun investigating how creating ad hoc self‐associations can modulate cognitive processing. In such studies participants are told to associate arbitrary stimuli (e.g., geometrical shapes) with various identities. Crucially, one of these identities is the participant themself. For example, a participant can be asked to associate a triangle with themself (through an instruction: “You are a triangle”), a square with a friend, and a circle with a stranger (Sui, He, & Humphreys, 2012; Sui, Rotshtein, & Humphreys, 2013). The results show that even an arbitrary association with the self leads to robust prioritization of self‐associated stimuli, as manifested by shorter reaction times and improved detection accuracy. In behavioral studies, the same procedure has been also used with bodily stimuli such as faces and avatar bodies, yielding similar effects (Mattan, Quinn, Apperly, Sui, & Rotshtein, 2015; Payne, Tsakiris, & Maister, 2017; Woźniak & Hohwy, 2020; Woźniak & Knoblich, 2019). At the neural level, several previous experiments investigated which brain areas are affected by creating such arbitrary self‐associations for abstract, symbolic stimuli (Lockwood et al., 2018; Sui et al., 2013; Sui, Liu, Mevorach, & Humphreys, 2015; Yankouskaya et al., 2017; Yankouskaya & Sui, 2021; Yin et al., 2021; Zhao, Uono, Li, Yoshimura, & Toichi, 2018) and found that midline cortical structures (especially the ventromedial prefrontal cortex) are critically involved in such tasks. However, to date, no neuroimaging study used this approach to investigate which brain areas represent bodily, rather than abstract, information associated with the self. This is a significant omission, as bodily self is arguably more basic than the conceptual self (Christoff et al., 2011; Gallagher, 2000; Gillihan & Farah, 2005; Woźniak, 2019), and also because, as mentioned before, previous research found that brain regions underpinning processing of bodily information rarely overlap with brain regions responsible for processing of abstract self‐related information (Gillihan & Farah, 2005; Hu et al., 2016; Qin et al., 2020).

The present study investigated, focusing on full‐body movements, which brain regions differentially represent self‐ versus other‐associated bodily information. Specifically, it was designed to address how the brain differentiates between seeing identical actions—that is, the same movements presented from a third‐person perspective—attributed to oneself or to other people. This stands in contrast to previous studies on self‐recognition of body movements, in which people were asked to recognize their real body movements (Bischoff et al., 2012; Jokisch, Daum, & Troje, 2006), and which hence targeted different cognitive processes. In the current study participants were presented with stimuli depicting different types of movements, presented as point‐light walkers (short animations presenting people performing an action, but devoid of any information about their physical body appearance). Critically, across different trials participants were told to attribute the same movements either to themselves, or to others, which serves to match familiarity between the conditions. We employed a working memory (WM) paradigm (delayed‐match‐to‐sample task) during which participants had to form and actively maintain the representation of a self or non‐self‐associated movement for several seconds. We used MVPA on functional magnetic resonance imaging (fMRI) data to detect brain areas that differently code memorized movement associated with the self or another person.

Previous research on self‐related processing suggest that it is mainly activity in three brain regions distinguishing whether a presented movement is associated with oneself or other identities. First, selective processing of self‐related information have been reported in frontal cortices that are also involved in action recognition and which are believed to be part of the human mirror‐neuron system, especially the inferior frontal gyrus (Molenberghs, Cunnington, & Mattingley, 2012). Second, the ventral medial prefrontal cortex has been found to play an important role in processing abstract self‐related information, such as one's name, self‐related adjectives, or arbitrary self‐associated stimuli (Hu et al., 2016; Sui et al., 2013; Yankouskaya et al., 2017). Finally, the temporal parietal junction and surrounding areas have been identified as being critically involved in self‐other distinction across a range of cognitive processes (Ganesh, van Schie, de Lange, Thompson, & Wigboldus, 2011; Hu et al., 2016; Quesque & Brass, 2019; Sui et al., 2013). Based on these reports, we were particularly interested in the involvement of these three regions in representing movements as self‐related. To test this, we included three experimental conditions, namely memorizing the movement of oneself, or of two different stranger identities. This design allowed us to test for distinguishable activation patterns evoked by the self‐associated and stranger‐associated movements, while we expected no differences between the two strangers.

## 2 METHODS

### 2.1 Participants

The inclusion criteria for participation were age over 18 and no psychiatric or neurological disorders. Twenty‐four volunteers participated in the study (age range: 21–40 years; *M* = 26.3, *SD* = 4.89; 13 females, 11 males). All participants were right‐handed. All participants had normal or corrected‐to‐normal vision. Informed consent was obtained from all participants before the start of the experiment according to procedures approved by the Freie Universität Berlin Ethics Committee.

### 2.2 Procedure

The experimental paradigm comprised a delayed match‐to‐sample working memory task employing retro‐cues associated with specific identities. Inside the MRI scanner, participants were first familiarized with the task while structural MRI data was acquired. Next, five experimental runs of 15 min 30 s each were conducted while functional MRI data were collected.

At the beginning of the experiment, participants learned which of three colors (red, green, blue) represents which one of three identities: You (the participant her‐ or him‐self), Pat (Patricia or Patrick—gender of the name was always matching with participant's gender), and Doc (a gender‐matched stranger who is a doctor). The labels “Pat” and “Doc” were chosen as indicators of two strangers' identities because they have the same length as the word “You,” they can be used to reflect both genders (the identity of the strangers were always instructed as of the same gender as the participant, to exclude potential effects of associations with a different gender‐identity than the own one), and each belongs to a different grammatical category ruling out potential confound effect of grammatical distinctiveness (Schäfer, Wentura, & Frings, 2017). The associations between colors and identities were presented in a written form on the instruction sheet which was given to each participant. The color‐identity associations were counterbalanced across participants using a Latin square scheme. Color‐identity associations were introduced to the task in order to avoid using labels as retro‐cues in the experimental task.

**FIGURE 1:**
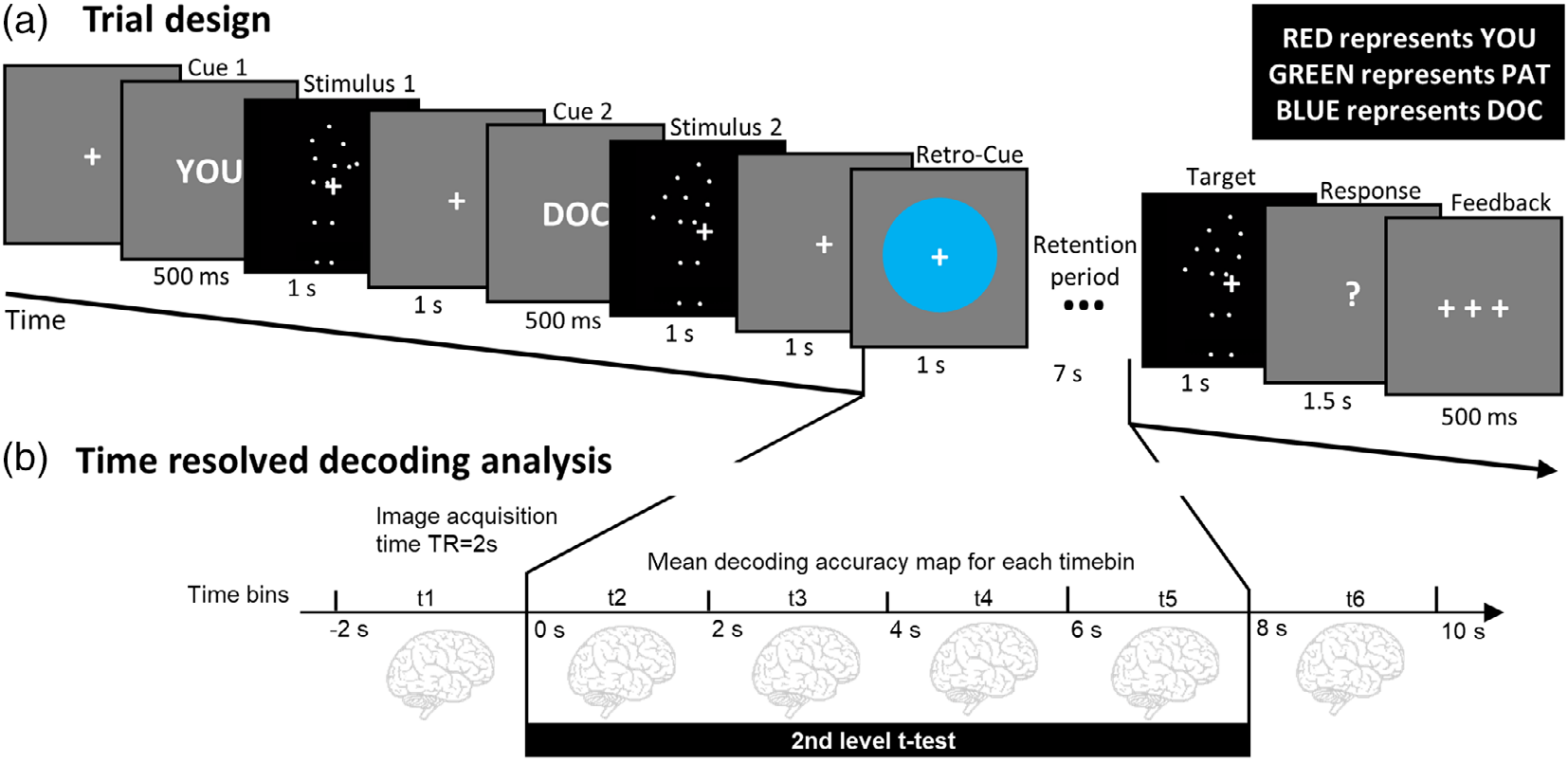
Experimental paradigm and analysis. (a) Before the experiment started participants were told which of three colors represent which identity (example given in upper right panel, assignment of colors was counterbalanced across participants). During fMRI scanning, participants performed a retro‐cue delayed match‐to‐sample working memory task. In each trial, two full‐body movement stimuli were presented and a consecutive retro‐cue indicated which of the two movements had to be retained for a 7‐s retention period. Finally, a target movement stimulus was presented and had to be judged whether it was the same or different from the memorized movement. A distinctive feature of this task was that each of two presented movements was associated with one of the three identities: Either with the participant oneself or with one of two strangers. The color retro‐cue indicated which movement should be memorized by referring to the identity of a person performing a movement. (b) As fMRI data acquisition was time‐locked to the start of the working memory retention period, it was possible to conduct time‐resolved decoding analysis. In short, whole‐brain searchlight classification analyses were performed on the data of each timebin, corresponding to separate functional volumes. We used a group‐level t‐contrast across timebins t2–t5 to test for above‐chance decoding accuracies

Figure 1 illustrates the procedure of the retro‐cue task used in the present study. In each trial participants had to memorize a biological motion stimulus, which was indicated to be either a movement of oneself or of another person. Each trial started with the presentation of two short animations of movements displayed as point‐light walkers (each 1,000 ms, separated by a 1,000 ms ISI, see section “Stimuli”). Before each stimulus the identity performing the motion was indicated by an identity‐cue: either “YOU” (self‐condition), “PAT”, or “DOC” (others‐conditions) for 500 ms (see Figure 1). For example, if the first cue was “PAT” then it indicated that the first movement was performed by Pat, and if the second cue was “Doc” then it conveyed the information that the second movement was performed by Doc. If one of the cues was “You” then participants were told to interpret the corresponding movement as if they were watching themselves from a third‐person perspective (for example on a video recording). After the presentation of stimuli, a retro‐cue instructed which of these two movements should be maintained for the working memory phase. The retro‐cue was either a red, blue, or green circle and indicated the identity (You, Pat, or Doc) whose movement had to be memorized, according to the previously learned color‐identity mappings. For example, if the associations were the same as presented in Figure 1a (upper right) then a blue retro‐cue told a participant to memorize Doc's movement, and if it was red then they were expected to memorize “Your” (participant's) movement. After a 7‐s retention period, a target movement was presented and participants' task was to decide if the target movement was identical (i.e., the same type of movement and presented from the same point of view) to the memorized movement. Reports were given via right‐hand middle and index finger button presses (yes/no–left/right balanced across participants).

Colors rather than labels were used as retro‐cues to avoid potential confounds related to label‐specific processing, as there exists evidence that self‐related labels are processed preferentially over other labels (Hu et al., 2016; Shi, Zhou, Han, & Liu, 2011; Woźniak & Knoblich, 2019; Woźniak, Kourtis, & Knoblich, 2018; Zhou et al., 2010). At the same time, familiar labels were used as cues indicating associations of each presented movement. Previous research found that the effect of self‐association is present only when associations between stimuli and identities are perceived as task‐relevant (Caughey et al., 2020; Woźniak & Knoblich, 2021). Therefore, we used labels as cues in our task to make remembering color‐identity associations necessary to complete the task and avoid the situation in which participants could use alternative strategies.

A target movement was only considered identical to the memorized movement if it was both the same type of movement (e.g., chopping, painting) and was presented from the same camera position, that is, it was identical in regard to low‐level visual features. Participants were required to make their responses within a time window of 1.5 s after the target movement offsets. Afterwards they were presented with feedback provided as “+ + +” (correct), “− − −” (incorrect), or “0 0 0” (missing response). The inter‐trial interval was either 1 or 3 s, balanced and randomly interleaved across trials.

Each run comprised 48 trials which were balanced with regards to: (a) which movement was retained in working memory, (b) whether the first or the second movement was retained, and (c) which identities were presented as cue 1 and cue 2. The target movement was identical to the memorized movement in 50% of the trials, a different type of movement in 25% (randomly selected) of the trials and in the remaining 25% it was the same type of movement, but presented from a different camera position. The remaining factors were counterbalanced across trials (which movement was presented as a target, which camera position was used for each movement, etc.).

### 2.3 Stimuli

The experiment was programmed and conducted using Matlab (version R2013 8.2). The Psychophysics Toolbox (version 3.0.10) was used for presentation of the experimental task, and Cogent 2000 Toolbox (developed by the Cogent 2000 team at the FIL and the ICN) was used to create log files.

Short movie clips presenting point‐light walkers illustrating eight different movements were chosen from the data set created by (Vanrie & Verfaillie, 2004). All stimuli portrayed full bodies performing movements with their hands while standing (chop, drink, mow, paint, peddle, spade, stir, sweep) from five different camera positions (frontal, 45° left, 90° left, 45° right, 90° right). Because original clips differed in length, only 1 s was selected from each of them. For each participant, four of these eight movement stimuli were randomly selected from our stimulus set, to minimize the potential of stimulus‐specific confounding effects. The assignment of movements to participants was pseudo‐random in a way that ensured that each movement type was used for an equal number of participants.

Visual stimuli were projected with an LCD projector (800 × 600, 60 Hz frame rate) onto a screen on the MR scanner's bore opening. Participants observed the visual stimuli via a mirror attached to the MR head coil from a distance of 110 ± 2 cm. Fixation cross and visual cues were presented in white on a grey background. All identity‐cues were presented with approx. 0.5° height and 1°; the radius of the retro‐cue was 0.2°. The size of point‐light walker stimuli was 3.2 × 4.3°.

### 2.4 Data acquisition and preprocessing

MRI data were acquired using a 3 T TIM Trio scanner (Siemens, Erlangen) at the Center of Cognitive Neuroscience Berlin at Freie Universität Berlin. A structural T1‐weighted MRI image was acquired (MPRAGE; 176 sagittal slices/3D acquisition; TR = 1,900 ms; TE = 2.52 ms; 1 × 1 × 1 mm^3^ voxel; flip angle = 90°; field of view = 256 mm) when participants were practicing the task during the practice run. Functional MRI data were acquired in five runs, each lasting 15 min 30 s. In each run, 463 images were acquired (T2*‐weighted gradient‐echo EPI; 37 slices; ascending order; 20% gap; whole brain; TR = 2,000 ms; TE = 30 ms; 3 × 3 × 3 mm^3^ voxel; flip angle = 70°; field of view = 192 mm; 64 × 64 matrix). Trial onsets as well as onsets of retro‐cues and retention periods were time‐locked to the onset of functional image acquisition allowing TR‐wise analysis (see Figure 1).

### 2.5 Data analysis

Preprocessing of the data and GLM analyses were conducted using SPM12 (Wellcome Trust Centre for Neuroimaging, Institute for Neurology, University College London, London, UK). Preprocessing of functional data for decoding analyses was limited to spatial realignment and temporal detrending (Macey, Macey, Kumar, & Harper, 2004), to preserve the spatiotemporal structure of data for the decoding analysis.

#### 2.5.1 Time‐resolved searchlight decoding

We used a time‐resolved multivariate searchlight decoding approach as applied in previous working memory studies (Christophel, Hebart, & Haynes, 2012; Kriegeskorte, Goebel, & Bandettini, 2006; Schmidt, Wu, & Blankenburg, 2017). We performed two independent analyses on the data, in order to determine brain areas that (1) carry information about the memorized movement as such (Movement Decoding: MD) and (2) the identity of the person performing a memorized movement (Identity Decoding: ID).

The analysis was performed as in a series of several similar WM decoding studies to make the current results comparable in the best possible way (e.g., Christophel, Cichy, Hebart, & Haynes, 2015; Schmidt et al., 2017; Schmidt & Blankenburg, 2018; Uluç, Velenosi, Schmidt, & Blankenburg, 2020; Wu et al., 2018). For this purpose we first fitted two independent subject‐level GLMs comprised of finite‐impulse‐response (FIR) regressors (high‐pass filter cut‐off: 200 s) modeling the time period from 2 s before the onset of the retro‐cue to 2 s after the WM retention period, corresponding to six FIR regressors of 2 s each (see Figure 1b). The first GLM comprised regressors for the three identities (You, Pat, or Doc). The second GLM modeled the retention activity with regressors for the different movements irrespective of the identity. This was possible, as the factors movement type and identity were orthogonal in our experimental design. The FIR GLM conducted for Identity Decoding resulted in 95 beta estimates (3 conditions × 6 time bins × 5 runs + 5 constants), and the FIR GLM for Movement Decoding yielded 125 beta estimates (4 stimuli × 6 time bins × 5 runs + 5 constants). Beta estimates were forwarded to the subsequent multivariate pattern analysis.

We used The Decoding Toolbox (TDT) (Hebart, Görgen, & Haynes, 2015), which provides an interface for applying machine learning algorithms to neuroimaging data using LIBSVM library (Chang & Lin, 2011). We combined MVPA with a searchlight routine (Kriegeskorte et al., 2006), which allowed us to conduct data‐driven analysis without any prior assumptions on the localization of information. The spatially‐unbiased searchlight MVPA was performed for each of the six time bins. In the searchlight analysis, a spherical region‐of‐interest (radius = 4 voxels) was moved voxel‐by‐voxel through the measured brain volume. At each position, we used Support Vector Machine (SVM) to test for the decodability of WM contents in a pair‐wise manner (linear kernel with constant regularization parameter *c* = 1). Beta estimates for each time bin were *z*‐scaled (normalized) across the samples for each voxel as implemented in TDT. The decoding analyses were performed using a fivefold, leave‐one‐run‐out cross‐validation decoding scheme across the five experimental runs. This procedure yields whole‐brain decoding accuracy maps for each subject and each time bin. Decoding accuracy maps were normalized to MNI space, using unified segmentation and spatially smoothed using a 5 mm FWHM Gaussian kernel, using SPM 12 routines. For the Movement Decoding analysis accuracy maps for each comparison between four movements (six comparisons per participant) were averaged to obtain one decoding accuracy map per timebin. For the Identity Decoding analysis accuracy maps for comparisons between the participant and other identities (You vs. Pat and You vs. Doc) were averaged to obtain one map of You vs. Others comparison per timebin. The accuracy maps for comparison Pat vs. Doc for each timebin were used as a control condition contrasting two stranger identities.

We obtained 6 accuracy maps for Movement Decoding (reflecting decoding in six time bins), and 12 accuracy maps for Identity Decoding: six maps reflecting decoding of self versus others (Pat and Doc), and six maps reflecting decoding of Pat versus Doc. Above chance decoding accuracy maps were entered into flexible‐factorial design group‐level analyses, which corresponds to SPMs realization of group‐level ANOVA designs (Gläscher & Gitelman, 2008). We ran three independent group‐level models: (1) Movement Decoding, (2) Identity Decoding: self versus others, and (3) Identity Decoding: Pat versus Doc. Within these group‐level models we computed t‐contrasts across the time‐bins of interest (2, 3, 4, and 5) against zero, corresponding to testing the mean decoding‐accuracy minus chance across these time‐bins against zero (see Figure 1). Results are reported at *p* < .05, family‐wise error (FWE) corrected for multiple comparisons, with a minimal cluster extent threshold of 50 voxels.

### 2.6 Control analysis: Decoding of non‐memorized stimulus

In order to test for the specificity of our main decoding analyses, we conducted control analyses to test for above chance decoding of the stimulus which was not retained for working memory (uncued stimulus). New FIR models were estimated which, due to the balanced experimental design, had the same amount of regressors, as each stimulus was equally often memorized as they were presented and not memorized. We performed analogous decoding analyses for the non‐memorized (1) Movement Decoding, (2) Identity Decoding: self versus others, and (3) Identity Decoding: Pat versus Doc.

#### 2.6.1 Univariate analysis

A univariate analysis was conducted in order to identify regions that exhibit increased activity when processing self‐ than other‐related movements. Preprocessing of functional MRI data comprised realignment, coregistration with the structural scan, normalization to MNI space using unified segmentation, smoothing with 5 mm FWHM Gaussian kernel, and temporal detrending (Macey et al., 2004). Data was entered into a standard SPM12 GLM analysis (High‐pass cut‐off: 128 s) including run‐constants to model potential data offsets between runs. Our first‐level design included 10 HRF convolved regressors per run to model for every trial: (1) the stimulus presentation phase before the retro‐cue; (2–4) the retro‐cues, for self‐, Pat‐, and Doc; (5–7) the WM retention period for self, Pat, and Doc; (8) the onset of the target stimulus; and (9,10) participants' responses for left‐ and right‐button presses. Subject‐level contrasts were computed for the self versus other retro‐cue and WM retention periods and tested against zero in a group‐level one‐sample *t*‐test. An analogous control analysis was conducted for the Pat versus Doc comparison. Results are reported at *p* < .05 FWE corrected on the cluster level.

## 3 RESULTS

### 3.1 Behavioral performance

Participants performed with a mean of 83.6% (*SD* = 5.7%) correct responses in the applied match‐to‐sample task (range: 70–94.2%). Testing for differences in performance levels (percentage of correct responses) with a one‐way repeated measured ANOVA with factor identity (self, Pat, Doc) revealed a significant main effect of identity (*F*(2,46) = 5.49, *p* = .007, partial *η* ^2^ = 0.19). Post hoc *t*‐tests showed that participants were significantly less accurate to recognize a self‐associated movement (*M* = 81.7%, *SD* = 5.4%) than a movement associated with Pat (*M* = 84.5%, *SD* = 6.3%, *t*(23) = 3.38, *p* = .008 Bonferroni‐corrected) or Doc (*M* = 84.4%, *SD* = 7.2%, *t*(23) = 2.68, *p* = .04 Bonferroni‐corrected), while the difference between Pat and Doc was not significant (*t*(23) = 0.1, *p* = 1 Bonferroni‐corrected).

In order to compare accuracies for different types of movements we conducted a linear mixed model (LMM) analysis. Conducting an LMM analysis was necessary in order to account for the crossed‐nested character of this data. The analysis did not reveal any significant differences between movements. We conducted an LMM analysis using Statsmodels Python API with dependent variable being accuracy (percentage of correct responses), each of eight movements used in the experiment as independent variables, and participant as a grouping factor. We did not find any significant differences between accuracies for memorizing different types of movements (all movements were compared against the movement with highest accuracy, i.e., chopping).

### 3.2 Movement decoding

**FIGURE 2:**
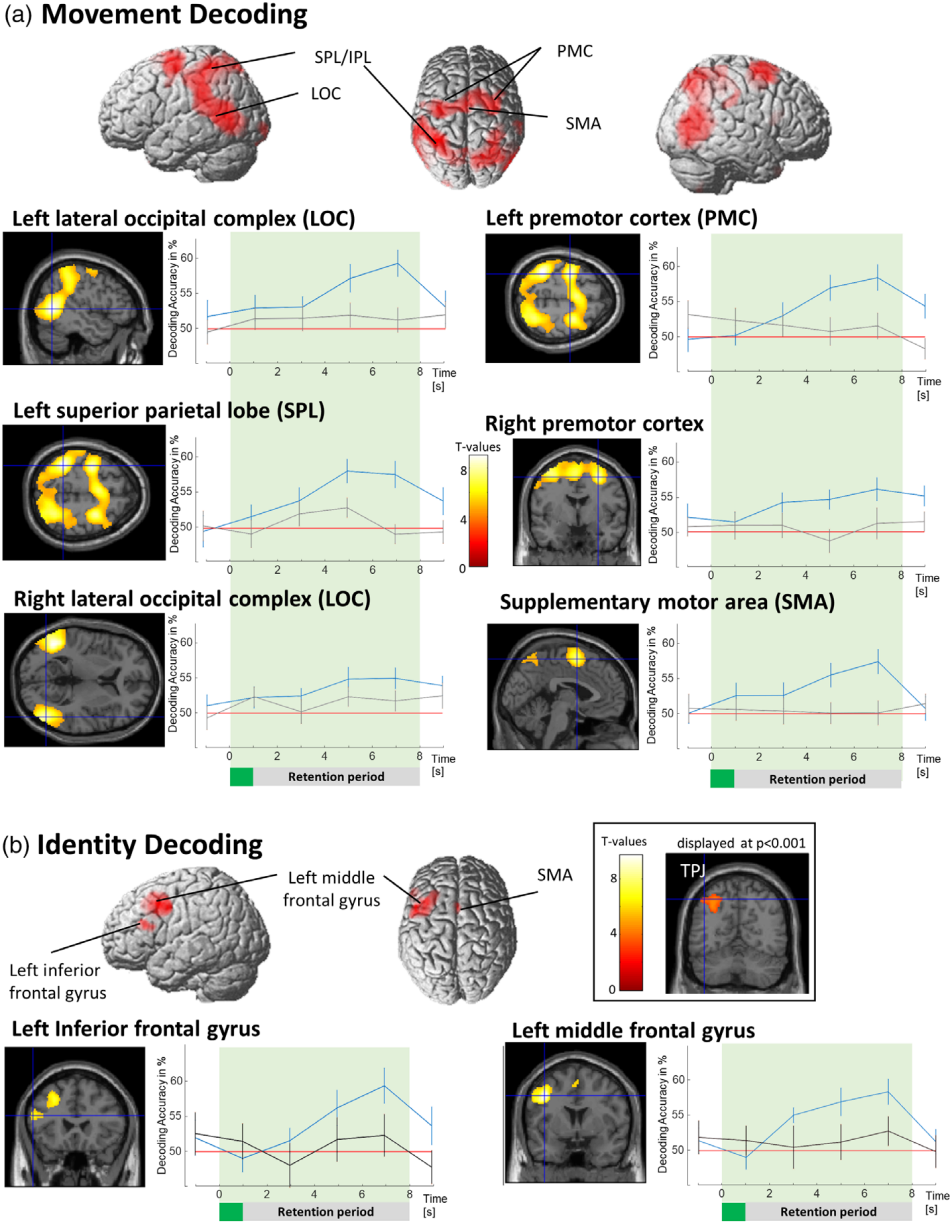
(a) Brain areas representing information about the type of movement held in working memory. Time‐courses of the descriptive evolution of decoding accuracies over the delay period are shown. The peak voxels were selected from the whole‐brain analysis, impeding further statistical assessment. Blue lines represent decoding accuracy from peak voxels for memorized movement. Grey lines represent decoding accuracy from the same areas for non‐memorized movement. The red line represents the 50% chance level of the decoding accuracy maps. (b) Brain areas representing information about whether the movement held in working memory is associated with the self versus others. Blue lines represent decoding accuracy from peak voxels between self and others as a factor of time. Black lines represent decoding accuracy from the same areas between two control (“other”) identities: Pat and Doc. All results displayed at *p* < .05, FWE corrected, error bars indicate SEM

To identify regions where brain activation relates to the movement presented in the stimulus, we computed a *t*‐contrast to test for above‐chance decoding accuracies during the WM retention. The results (at *p* < .05 FWE, min. cluster size = 50) are displayed in Table 1 and Figure 2. The largest cluster encompasses a big part of the parietal cortex, as well as occipital and temporal cortices (all bilaterally), with peaks in the left (posterior) middle temporal gyrus, left superior parietal lobe, and right inferior occipital gyrus. The second‐largest cluster spans the bilateral precentral gyrus as well as the supplementary motor area.

**TABLE 1:** Regions with above‐chance decoding accuracy for movement decoding (a) and identity decoding (b) during the retention period at *p* < .05, FWE corrected

		Peak MNI coordinates				
Cluster size	Anatomical region	*x*	*y*	*z*	*z*‐score	*T*
(a) Movement decoding						
19,015	Left middle temporal gyrus	−48	−60	4	7.82	9.03
	Left superior parietal lobule	−36	−46	56	7.77	8.95
	Right inferior occipital gyrus	50	−70	6	7.19	8.10
5,587	Left precentral gyrus	−34	−8	58	7.43	8.44
	Right precentral gyrus	30	−2	54	7.11	7.99
	Left supplementary motor cortex	−2	2	58	6.81	7.58
135	Right temporal pole	20	20	−38	6.40	7.04
305	Left inferior occipital gyrus	−30	−94	−22	5.54	5.95
313	R inferior frontal gyrus/white matter	36	16	20	5.03	5.34
185	Left posterior cingulate gyrus	−8	−42	−36	4.86	5.13
293	Right supramarginal gyrus	64	−28	36	4.79	5.05
61	Right exterior cerebellum	36	−62	−56	4.61	4.85
(b) Identity decoding						
1,170	Left middle frontal gyrus	−40	4	40	7.46	9.73
	Left inferior frontal gyrus, pars opercularis	−54	24	20	5.33	5.99
68	Left supplementary motor area	−2	14	54	5.21	5.77

*Note*: Only clusters encompassing at least 50 voxels are displayed.

Time courses of decoding accuracies for the identified regions are displayed in Figure 2. The delayed build‐up of decoding accuracies along with the leveling off until the end of the retention period indicate that the decoding results are specific to a WM representation and do not stem from mere perceptual processing (see also control analyses).

### 3.3 Identity decoding

In order to localize brain areas representing identity‐related information, we computed two decoding analyses: (1) Self versus Other identities, and (2) Pat versus Doc. Brain regions representing self‐related information should show above‐chance level decoding accuracies only in (1), where activation patterns of a self‐related condition are compared with activation pattern elicited by Others. In turn, regions coding self‐related information are assumed to not show above‐chance decoding accuracies when activation pattern of two Other persons are compared. Thereby regions revealed in (1) and not in (2) code not only the difference between two identities, but also the difference between movements encoded for oneself and movements encoded for other persons. A corresponding *t*‐contrast of decoding accuracies against chance‐level across the WM retention period (*p* < .05, FWE corrected, min. cluster size = 50) revealed two clusters for self versus others: One cluster spanning the left middle frontal gyrus and the left inferior frontal gyrus, pars opercularis, and a smaller cluster in the left supplementary motor area (Figure 2, Table 1).

Following our a priori hypothesis we inspected the data also at a lower threshold to check for above change decoding accuracy in the TPJ and vmPFC, which might have been missed due to conservative FWE correction threshold. Indeed, this revealed a cluster in the TPJ, more specifically angular gyrus (*p* < .001, FWE‐corrected on the cluster level; peak coordinates: *x* = −30, *y* = −64, *z* = 48, size = 643 voxels, *z* = 436). No above chance decoding was found in the vmPFC.

Testing for above‐chance decoding between the two Other‐identities Pat versus Doc yielded no significant clusters. This suggests that the regions revealed in the Self versus Other decoding are specific to the coding of self‐related information.

### 3.4 Control analyses: Decoding of non‐memorized content

We ran three control decoding analyses to test for the specificity of the main results: the (1) Non‐memorized Movement Decoding, (2) Non‐memorized Identity Decoding: self versus others, and (3) Non‐memorized Identity Decoding: Pat versus Doc. These analyses contained the same amount of data and the same processing steps as the three main analyses but tested if information can be decoded on the corresponding non‐memorized stimuli. No significant clusters of activation were found in either of the analyses.

Additionally, we tested for potential interaction effects between movement and identity decoding. For this purpose, we performed an additional analysis decoding for differences between the movements (Movement decoding). However, in contrast to the main Movement decoding analysis, here the trials of one movement type were split according to whether the movement was associated with self, Pat, or Doc. We then tested with a second level design for differences in decoding accuracies for Movement decoding between Self versus Pat, Self versus Doc, and Pat versus Doc. These analyses did not reveal any significant differences, even at a reduced threshold (*p* < .001, uncorrected) indicating no relevant interaction.

### 3.5 Univariate analysis

**FIGURE 3:**
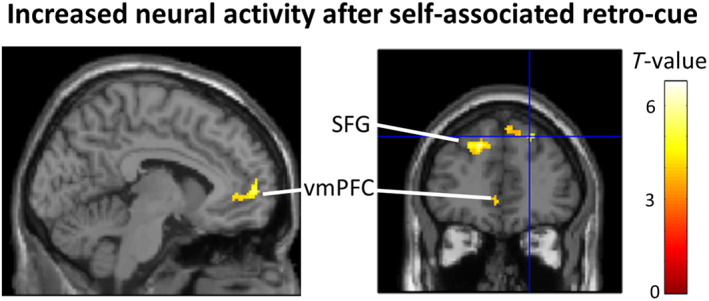
Presentation of a self‐associated retro‐cue led to increased activity in the ventral medial prefrontal cortex and bilaterally in the superior frontal gyri. The *p* < .05 FWE‐corrected on the cluster level

Testing in an assumption‐free manner throughout the whole brain for the self > other contrast of the retro‐cue revealed activation clusters in the left vmPFC (*x* = −6, *y* = 58, *z* = 4, cluster size = 113 voxels, *z*‐score = 3.88, *T*‐score = 4.67; Figure 3), the left superior frontal gyrus (*x* = −20, *y* = 34, *z* = 42, cluster size = 231 voxels, *z*‐score = 4.44, *T*‐score = 5.66) and in the right superior frontal gyrus (cluster1: *x* = 22, *y* = 42, *z* = 44, cluster size = 150 voxels, *z*‐score = 4.56, *T*‐score = 5.89), at *p* < .05 FWE‐corrected on the cluster level (clusters did not survive *p* < .05 FWE correction for the whole brain). Contrasting self versus other for the WM retention period did not reveal any significant clusters.

Testing for differences between the two stranger‐identity conditions (Pat and Doc, in both directions) did not reveal any significant clusters neither for the retro‐cue, nor throughout the working memory retention period (*p* < .001 uncorrected).

## 4 DISCUSSION

We conducted an fMRI study and used MVPA to test which brain areas code information about whether a memorized movement was represented in WM as performed by oneself or by someone else. In comparison to previous studies, we used MVPA to obtain information maps, reflecting where in the brain the patterns of activation allow to distinguish between the representation of one or another content of working memory. Thereby our study did not only investigate the general involvement of brain regions, but we are able to show that these regions exhibit codes that are specific to the mental representation of a specific working memory content. First, we tested for the brain regions in which activation patterns relate to the retention of specific movements (e.g., chopping, painting, etc.: Movement Decoding). We found regions, related to higher‐order perceptual and multimodal integration processing of movements, such as the superior temporal sulcus, middle temporal cortex, extrastriate body area, intraparietal sulcus, as well as supplementary and premotor/motor cortices. Testing for brain regions that exhibit distinct activation patterns for movements associated with oneself as compared with those associated with another identity (Identity Decoding), our analyses revealed the left middle frontal gyrus, inferior frontal gyrus, and supplementary motor area. Finally, we found increased activation in the vmPFC in response to the presentation of a self‐associated retro‐cue providing further support for previous findings from (Sui et al., 2013; Yankouskaya et al., 2017), as well as in bilateral SFG, however not during the WM retention period.

### 4.1 Movement decoding

The introduction of machine learning techniques, especially multivoxel pattern analysis to neuroimaging, improved the sensitivity of analyses to detect fine differences between activation patterns. This has been particularly influential for WM research (Christophel, Klink, Spitzer, Roelfsema, & Haynes, 2017; Riggall & Postle, 2012). The conventional univariate analysis is limited to the detection of the overall mean activation change in a given brain region between two conditions. This approach is often not suitable to assess the neural coding of specific WM contents as the overall activation elicited by different WM contents are often indistinguishable. In contrast, MVPA allows to test if the retention of different WM contents (e.g., stimulus 1 or stimulus 2) lead to different activation pattern within a brain regions. Thereby the results of MVPA searchlight analyses can be interpreted as information maps rather than activation maps. This means that the regions revealed by our analysis relate to the actual content representation of WM. Thereby the contribution of these regions to realize a mental WM representation is considered to go beyond The differences in IFG, MFG, and SMA general support functions, such as attentional mechanisms or cognitive control contributions to WM. These regions are thought to realize the representation of a WM content. Multiple WM MVPA studies indicate that the brain regions that exhibit activity related to specific WM contents partly overlap with the brain regions involved in the perceptual processing of corresponding stimulus material (Lee & Baker, 2016). It has been suggested that the amount of overlap is determined by the degree of abstractness in which contents are retained, for example, in a rather sensory format, like imagining the visual appearance of a shape, or in a rather symbolic or language format, like used when rehearsing a telephone number (Christophel et al., 2017). Our movement decoding analysis extends this literature, by testing what brain regions exhibit codes for the retention of more complex and more naturalistic material, namely specific movements (Galvez‐Pol, Forster, & Calvo‐Merino, 2020). Indeed, the regions revealed by our analysis partly overlap with regions known for perceptual processing of biological motion and action observation (Caspers, Zilles, Laird, & Eickhoff, 2010; E. Grossman et al., 2000; E. D. Grossman & Blake, 2002; Lu et al., 2016; Pelphrey, Morris, Michelich, Allison, & McCarthy, 2005; Saygin, 2007a; Saygin, Wilson, Hagler, Bates, & Sereno, 2004; Vaina, Solomon, Chowdhury, Sinha, & Belliveau, 2001; van Kemenade, Muggleton, Walsh, & Saygin, 2012).

Finding only a small cluster in early visual areas indicates that the mere visual appearance of the stimulus material plays a minor role for the given WM representations, in contrast to the retention of static visual stimulus material (e.g., Albers, Kok, Toni, Dijkerman, & De Lange, 2013; Christophel et al., 2012). As expected, our analysis revealed bilateral clusters in the lateral occipital cortex (LOC), including the motion‐sensitive human middle temporal (hMT) region and the extrastriate body area (EBA), which have been shown to be involved in the visual processing of human body parts (Limanowski & Blankenburg, 2016). The identified cluster extends to the posterior superior temporal sulcus (pSTS), which has been found to be involved in social perception, including being sensitive to biological motion and goal‐directed actions (Pelphrey et al., 2005; Pitcher & Ungerleider, 2020; Saygin, 2007b; Shultz, Lee, Pelphrey, & McCarthy, 2011; Vaina et al., 2001). Superior‐ and intraparietal lobe regions (SPL/IPL) have been found in visual and tactile WM tasks where spatial information was retained, and are well known for their involvement in the remapping of spatial coordinate systems between allocentric and egocentric perspectives, particularly important during movements (Christophel et al., 2012; Heed, Buchholz, Engel, & Röder, 2015; Schmidt & Blankenburg, 2018). Finally, as expected, our analysis revealed pre‐ and supplementary motor areas. These regions have been found to contribute to different types of WM representations (Schmidt et al., 2017; Uluç, Schmidt, Wu, & Blankenburg, 2018), where they are thought to reflect attentional top‐down mechanisms of mental rehearsal processes (compare: Schmidt, Schröder, Reinhardt, & Blankenburg, 2020). These regions are also well known for their contributions to action planning and motor coordination. Therefore, they most likely reflect the mental simulation of the memorized movement (Rizzolatti & Sinigaglia, 2010, but see also: Cook, Bird, Catmur, Press, & Heyes, 2014).

Taken together, the identified regions partly overlaps with regions involved in perceptual processing of dot‐motion movement‐stimuli (Lu et al., 2016; Sokolov et al., 2018) and regions involved in mental simulation of movements, and replicates findings from previous studies (Galvez‐Pol, Forster, & Calvo‐Merino, 2020).

### 4.2 Identity decoding

The main goal of our study was to test in which brain area's activation pattern allows us to distinguish whether a memorized full‐body movement was represented as a movement performed by oneself or performed by someone else. In contrast to previous research, we did not investigate self‐recognition or memorization of one's real movements (Bischoff et al., 2012; Jokisch et al., 2006), but coding of movements only attributed to oneself. It means that our study did not target the same processes of action recognition based on visual and motor familiarity as previous research. Our results demonstrate that even an arbitrary movement, if it is interpreted as one's own movement, induces different brain activation patterns than a movement not associated with oneself. We found above‐chance decoding accuracy in one cluster spanning the left middle frontal gyrus and the left inferior frontal gyrus pars opercularis, and a second cluster in the left supplementary motor area. All of these areas (especially the IFG pars opercularis) have previously been identified to be active during action observation and imitation (Caspers et al., 2010; Johnson‐Frey et al., 2003; Molnar‐Szakacs, Iacoboni, Koski, & Mazziotta, 2005), as well as retention in WM of bodily actions (Galvez‐Pol, Calvo‐Merino, & Forster, 2020). Furthermore, there is evidence that IFG pars opercularis and MFG constitute parts of the human mirror neuron system, as indicated by ALE‐meta‐analysis by (Molenberghs et al., 2012). The differences in IFG, MFG, and SMA were revealed by our MVPA, while the corresponding univariate comparison did not reveal differences. This shows that our results do not simply reflect that the activity was greater in these areas, when self‐ than other‐associated movements were retained in WM. Instead, they suggest that neural codes, or the activation pattern within these areas, differ when one observes a movement that is represented as “myself” versus “other”, which can be detected by the higher sensitivity of MVPA as compared with univariate analyses. Importantly, we found these differences in the motor areas, even though participants could have finished the task without representing the movements as self‐related and by just memorizing the labels. However, if that were the case then we would observe no differences between conditions. The fact that we found selective coding of self‐associated movements in motor areas shows that participants completed the task as required, what is further supported by their reports after finishing the study.

All movements used in our study were displayed from the third person (allocentric) perspective, that is, how we usually perceive other people. A situations in which we observe our bodies acting from that perspective is when we see ourselves in a mirror or on a video. However, in these two kinds of situations, we observe ourselves performing movements that we already know from experience—that is, we performed them when they were recorded (in case of a recently recorded video), or that we are performing right now (in case of a mirror). It has been found that activity of the mirror neurons network, including the IFG, is increased when observing actions that are familiar to us (Calvo‐Merino, Glaser, Grèzes, Passingham, & Haggard, 2004; Calvo‐Merino, Grèzes, Glaser, Passingham, & Haggard, 2006; Cross, Hamilton, & Grafton, 2006; Cross, Hamilton, Kraemer, Kelley, & Grafton, 2009). However, in our study, no such effect could directly account for the results, because our design excluded the effect of familiarity of the actions associated with each identity, because for every participant the same actions were equally often associated with all identities across trials. Therefore, our findings in the IFG do not simply reflect motor familiarity, instead they reflect in a well‐controlled manner whether a seen action is represented as self‐ or other‐related.

The second region we expected to be modulated by self‐association was the TPJ. With a more liberal significance threshold we found that activity in the left angular gyrus, which constitutes a dorsal posterior part of the TPJ, differentiated between maintaining representations of self‐ and other‐associated movements. Bilateral TPJ has been suggested as an area that is responsible for implementing domain‐general self‐other distinction, as shown by a large variety of tasks involving self‐body perception, self‐agency, and mindreading (Quesque & Brass, 2019). Some research points to the role of the left TPJ in third‐person self‐representation (Ganesh et al., 2011), but more research is needed to fully establish this link. Previous studies on perception of actions performed by self or others that have identified TPJ activity mainly used videos and animations of participants' real movements—stimuli that were portraying highly familiar movement patterns (e.g., Bischoff et al., 2012; Macuga & Frey, 2011; Sugiura et al., 2006). These studies typically argued that the role of TPJ in self‐other distinction relies on detecting motor familiarity, and specifically on comparing predicted and observed body movements. However, because all animations used in our study reflected kinematics that were equally unfamiliar to participants (none of them was based on their real kinematics), our results support the view that the TPJ's role in representing the distinction between self and others is more domain‐general as advocated by Quesque and Brass (2019), and is not limited to a mechanisms that rely on familiarity of observed movements.

In contrast to previous studies on processing of movements attributed to oneself and others, in our study we did not use participants' real movements. Nevertheless, we found the effects of self‐attribution in similar brain areas to the ones that have been reported in studies using familiar self‐related bodily stimuli (IFG, MFG, TPJ). However, our results showed predominantly left‐lateralized effects, while previous reports on self‐related processing usually show right‐lateralized effects (Devue et al., 2007; Morita, Asada, & Naito, 2020), with less frequent reports of left‐lateralized effects. This difference in lateralization might be caused by familiarity. Indeed, the right hemisphere is more strongly implicated in cognitive processing of familiarity (Cross et al., 2009; Gainotti, 2007), meaning that right‐lateralization effects from previous studies might be driven by higher familiarity of self‐related than other‐related stimuli. However, more work is needed and there might be alternative explanation of the lateralization effects. For example, Ganesh et al. (2011), reported on neural correlates of self‐identification with a computer game avatars, and found increased avatar‐referential activity selectively in the left angular gyrus (Ganesh et al., 2011). The authors interpret their finding by suggesting that the left hemisphere might be more involved in self‐identification from the third‐person perspective (like in our study), while the right hemisphere dominates identification from the first‐person perspective.

The fact that our study involved unfamiliar stimuli might have had one more consequence: we found that self‐attributed movements led to different pattern of neural responses as uncovered by MVPA, but not to an overall increase of activity in these areas. This might suggests that the univariate results from previous studies could have been to certain extent strengthened by increased familiarity of presented stimuli.

Surprisingly, in our behavioral data we found that participants performed slightly worse in the forced‐choice task at the end of the WM delay period, when memorizing self‐associated movements than when they were memorizing other‐associated movements. Previous work has shown that self‐related material is processed in a privileged way and one could therefore expect a higher performance for WM of self‐related material also in our study (Sui & Humphreys, 2015; Symons & Johnson, 1997). However, previous studies showing higher accuracy for processing of arbitrarily self‐associated information used mostly abstract stimuli. This shows that associating arbitrary abstract information with the self does not lead to interference with one's conceptual self that would cause decrease in task performance. One interpretation that could explain why we found an opposite effect would be that the unfamiliar movements that had to be actively associated with oneself might cause interference with sensorimotor knowledge about one's real body dynamics. Therefore, the WM representation might have been influenced by one's own sensorimotor body representation underpinning the bodily self (De Vignemont, 2010, 2018; Dewey & Knoblich, 2016; Gallagher, 2005; Kanayama & Hiromitsu, 2021; Schwoebel & Coslett, 2005), which would make it more difficult to match the memorized movement with the comparison stimulus. This would also explain why previous reports did not find similar interference when participants were told to self‐identify with avatar faces (Payne et al., 2017; Woźniak et al., 2018; Woźniak & Hohwy, 2020)—static images of faces do not map onto sensorimotor bodily self representation like the biological motion does. This difference in performance should not have affected the main neuroimaging results, as a control analysis modeling only the correct trials confirmed above chance decoding in the three reported regions.

We did not find distinctive representations of self, in terms of differentiable activation patterns detectable by MVPA, during the WM retention period in one area in which we expected to find them: in vmPFC. Previous studies found that aspects of the conceptual self (self‐related adjectives, autobiographical memories, etc.) are usually associated with increased activity in midline structures (Qin et al., 2020; Qin & Northoff, 2011): precuneus and vmPFC, the latter of which is often regarded as the central node for self‐related processing (for a discussion see: D'Argembeau, 2013; Martinelli et al., 2013; Northoff et al., 2006). However, studies which investigated aspects of the self related to one's body, rather than personality, rarely find modulation of activity in the vmPFC or other midline structures (Gillihan & Farah, 2005; Hu et al., 2016; Legrand & Ruby, 2009; Qin et al., 2020). A possible interpretation of these results is that midline structures, in particular vmPFC, are more relevant for abstract and conceptual (e.g., semantic) information about the self than for the less abstract and more naturalistic bodily information associated with the self used in the study at hand.

### 4.3 Univariate results

In order to test if we can replicate previous reports on activation differences in vmPFC, when self‐related or non‐self‐related cues are processed, we additionally computed a corresponding univariate contrast for our data. In contrast to the multivariate decoding analysis which reveals differences between patterns of activation, in univariate analyses, which detect overall activity increases or decreases in a region, we did find modulation of activation in the vmPFC. Stronger retro‐cue induced activation was found for self‐associated retro‐cues as compared with non‐self related cues. In our study, the retro‐cue displayed one of the colors that prior to the experiment were associated with the self or the other identities. As such, it served the function of an abstract symbol referring to the self or others, in the same way as geometrical shapes that have been used in previous fMRI research on processing of arbitrary self‐associated stimuli (self‐prioritization effect: Sui et al., 2013; Yankouskaya et al., 2017, see also: Lockwood et al., 2018; Yin et al., 2021). Moreover, the specific vmPFC cluster detected in our MVPA analysis directly overlapped with the cluster found in previous studies, investigating univariate activation differences for processing of self‐associated shapes (Sui et al., 2013). This pattern of results replicates previous findings and support the interpretation that vmPFC is primarily associated with self‐related processing of conceptual aspects of the self, and not necessarily bodily aspects. Moreover, the finding that self‐association is related to increased activation in vmPFC after presentation of the retro‐cue, but not throughout the whole retention period, suggests that vmPFC is involved in selecting self‐associations for retention in working memory (Myers, Stokes, & Nobre, 2017), but not in maintaining them. This interpretation is consistent with previous research on the influence of self‐relatedness on memory, which indicates that vmPFC is involved in encoding and retrieval, but not maintenance of self‐related information (Kalenzaga et al., 2015; Leshikar & Duarte, 2012; Philippi, Duff, Denburg, Tranel, & Rudrauf, 2012; Yaoi, Osaka, & Osaka, 2015).

## 5 CONCLUSIONS

We conducted a study in which we tested which brain areas display different multivoxel activation patterns depending on whether a memorized action (seen from the third‐person perspective) is represented as being performed by oneself or by someone else. Crucially and in contrast to previous studies investigating this topic, as the same movements were associated with the self and with other identities in different trials, our study design rendered confounding influence of motor and perceptual familiarity unlikely. Our findings indicate that self‐associated movements are differentially represented during the working memory retention period through left hemispheric activation patterns in frontal areas responsible for action recognition and motor mirroring (MFG, IFG, and SMA). When testing for univariate activation differences, we further found increased activity in the vmPFC directly after the presentation of the retro‐cue that instructed to recall a self‐associated movement but no difference across the working memory retention period. This suggests that the influence of the vmPFC in our task might be limited to selecting a self‐associated movement for working memory, and not for maintaining it in working memory.

## Data Availability

The raw data generated during the current study are not publicly available due to data protection laws, but normalized data and the used scripts are available from the corresponding author on request.

## References

[hbm25879-bib-0001] Albers, A. M. , Kok, P. , Toni, I. , Dijkerman, H. C. , & De Lange, F. P. (2013). Shared representations for working memory and mental imagery in early visual cortex. Current Biology, 23(15), 1427–1431. 23871239 10.1016/j.cub.2013.05.065

[hbm25879-bib-0002] Baumeister, R. F. (1999). The self in social psychology. Psychology Press.

[hbm25879-bib-0003] Bischoff, M. , Zentgraf, K. , Lorey, B. , Pilgramm, S. , Balser, N. , Baumgartner, E. , … Munzert, J. (2012). Motor familiarity: Brain activation when watching kinematic displays of one's own movements. Neuropsychologia, 50(8), 2085–2092. 22609578 10.1016/j.neuropsychologia.2012.05.009

[hbm25879-bib-0004] Blanke, O. (2012). Multisensory brain mechanisms of bodily self‐consciousness. Nature Reviews. Neuroscience, 13(8), 556–571. 10.1038/nrn3292 22805909

[hbm25879-bib-0005] Blanke, O. , Slater, M. , & Serino, A. (2015). Behavioral, neural, and computational principles of bodily self‐consciousness. Neuron, 88(1), 145–166. 10.1016/j.neuron.2015.09.029 26447578

[hbm25879-bib-0006] Calvo‐Merino, B. , Glaser, D. E. , Grèzes, J. , Passingham, R. E. , & Haggard, P. (2004). Action observation and acquired motor skills: An FMRI study with expert dancers. Cerebral Cortex, 15(8), 1243–1249. 15616133 10.1093/cercor/bhi007

[hbm25879-bib-0007] Calvo‐Merino, B. , Grèzes, J. , Glaser, D. E. , Passingham, R. E. , & Haggard, P. (2006). Seeing or doing? Influence of visual and motor familiarity in action observation. Current Biology, 16(19), 1905–1910. 17027486 10.1016/j.cub.2006.07.065

[hbm25879-bib-0008] Caspers, S. , Zilles, K. , Laird, A. R. , & Eickhoff, S. B. (2010). ALE meta‐analysis of action observation and imitation in the human brain. NeuroImage, 50(3), 1148–1167. 20056149 10.1016/j.neuroimage.2009.12.112 PMC4981639

[hbm25879-bib-0009] Caughey, S. , Falbén, J. K. , Tsamadi, D. , Persson, L. M. , Golubickis, M. , & Macrae, C. N. (2020). Self‐prioritization during stimulus processing is not obligatory. Psychological Research, 85(2), 503–508. 31919569 10.1007/s00426-019-01283-2 PMC7900024

[hbm25879-bib-0010] Chang, C.‐C. , & Lin, C.‐J. (2011). LIBSVM: A library for support vector machines. ACM Transactions on Intelligent Systems and Technology (TIST), 2(3), 27.

[hbm25879-bib-0011] Christoff, K. , Cosmelli, D. , Legrand, D. , & Thompson, E. (2011). Specifying the self for cognitive neuroscience. Trends in Cognitive Sciences, 15(3), 104–112. 10.1016/j.tics.2011.01.001 21288760

[hbm25879-bib-0012] Christophel, T. B. , Cichy, R. M. , Hebart, M. N. , & Haynes, J.‐D. (2015). Parietal and early visual cortices encode working memory content across mental transformations. NeuroImage, 106, 198–206. 25463456 10.1016/j.neuroimage.2014.11.018

[hbm25879-bib-0013] Christophel, T. B. , Hebart, M. N. , & Haynes, J.‐D. (2012). Decoding the contents of visual short‐term memory from human visual and parietal cortex. Journal of Neuroscience, 32(38), 12983–12989. 22993415 10.1523/JNEUROSCI.0184-12.2012 PMC6621473

[hbm25879-bib-0014] Christophel, T. B. , Klink, P. C. , Spitzer, B. , Roelfsema, P. R. , & Haynes, J.‐D. (2017). The distributed nature of working memory. Trends in Cognitive Sciences, 21(2), 111–124. 28063661 10.1016/j.tics.2016.12.007

[hbm25879-bib-0015] Cook, R. , Bird, G. , Catmur, C. , Press, C. , & Heyes, C. (2014). Mirror neurons: From origin to function. Behavioral and Brain Sciences, 37(2), 177–192. 24775147 10.1017/S0140525X13000903

[hbm25879-bib-0016] Cross, E. S. , Hamilton, A. F. d. C. , & Grafton, S. T. (2006). Building a motor simulation de novo: Observation of dance by dancers. NeuroImage, 31(3), 1257–1267. 16530429 10.1016/j.neuroimage.2006.01.033 PMC1821082

[hbm25879-bib-0017] Cross, E. S. , Hamilton, A. F. D. C. , Kraemer, D. J. , Kelley, W. M. , & Grafton, S. T. (2009). Dissociable substrates for body motion and physical experience in the human action observation network. European Journal of Neuroscience, 30(7), 1383–1392. 19788567 10.1111/j.1460-9568.2009.06941.x

[hbm25879-bib-0018] D'Argembeau, A. (2013). On the role of the ventromedial prefrontal cortex in self‐processing: The valuation hypothesis. Frontiers in Human Neuroscience, 7, 372. 23847521 10.3389/fnhum.2013.00372 PMC3707083

[hbm25879-bib-0019] De Vignemont, F. (2010). Body schema and body image—Pros and cons. Neuropsychologia, 48(3), 669–680. 19786038 10.1016/j.neuropsychologia.2009.09.022

[hbm25879-bib-0020] De Vignemont, F. (2018). Mind the body: An exploration of bodily self‐awareness. Oxford University Press.

[hbm25879-bib-0021] Devue, C. , Collette, F. , Balteau, E. , Degueldre, C. , Luxen, A. , Maquet, P. , & Brédart, S. (2007). Here I am: The cortical correlates of visual self‐recognition. Brain Research, 1143, 169–182. 17306235 10.1016/j.brainres.2007.01.055

[hbm25879-bib-0022] Dewey, J. A. , & Knoblich, G. (2016). Representation of self versus others' actions. In S. S. Obhi & E. S. Cross (Eds.), Shared representations: Sensorimotor foundations of social life (p. 351). SAGE Publications.

[hbm25879-bib-0023] Gainotti, G. (2007). Face familiarity feelings, the right temporal lobe and the possible underlying neural mechanisms. Brain Research Reviews, 56(1), 214–235. 17822771 10.1016/j.brainresrev.2007.07.009

[hbm25879-bib-0024] Gallagher, S. (2000). Philosophical conceptions of the self: Implications for cognitive science. Trends in Cognitive Sciences, 4(1), 14–21. 10637618 10.1016/s1364-6613(99)01417-5

[hbm25879-bib-0025] Gallagher, S. (2005). How the body shapes the mind. Clarendon Press.

[hbm25879-bib-0026] Galvez‐Pol, A. , Calvo‐Merino, B. , & Forster, B. (2020). Revealing the body in the brain: An ERP method to examine sensorimotor activity during visual perception of body‐related information. Cortex, 125, 332–344. 32120169 10.1016/j.cortex.2020.01.017

[hbm25879-bib-0027] Galvez‐Pol, A. , Forster, B. , & Calvo‐Merino, B. (2020). Beyond action observation: Neurobehavioral mechanisms of memory for visually perceived bodies and actions. Neuroscience & Biobehavioral Reviews, 116, 508–518. 32544541 10.1016/j.neubiorev.2020.06.014

[hbm25879-bib-0028] Ganesh, S. , van Schie, H. T. , de Lange, F. P. , Thompson, E. , & Wigboldus, D. H. (2011). How the human brain goes virtual: Distinct cortical regions of the person‐processing network are involved in self‐identification with virtual agents. Cerebral Cortex, 22(7), 1577–1585. 21917741 10.1093/cercor/bhr227

[hbm25879-bib-0029] Gillihan, S. J. , & Farah, M. J. (2005). Is self special? A critical review of evidence from experimental psychology and cognitive neuroscience. Psychological Bulletin, 131(1), 76–97. 15631554 10.1037/0033-2909.131.1.76

[hbm25879-bib-0030] Gläscher, J. , & Gitelman, D. (2008). Contrast weights in flexible factorial design with multiple groups of subjects.

[hbm25879-bib-0031] Grossman, E. , Donnelly, M. , Price, R. , Pickens, D. , Morgan, V. , Neighbor, G. , & Blake, R. (2000). Brain areas involved in perception of biological motion. Journal of Cognitive Neuroscience, 12(5), 711–720. 11054914 10.1162/089892900562417

[hbm25879-bib-0032] Grossman, E. D. , & Blake, R. (2002). Brain areas active during visual perception of biological motion. Neuron, 35(6), 1167–1175. 12354405 10.1016/s0896-6273(02)00897-8

[hbm25879-bib-0033] Hebart, M. N. , Görgen, K. , & Haynes, J.‐D. (2015). The decoding toolbox (TDT): A versatile software package for multivariate analyses of functional imaging data. Frontiers in Neuroinformatics, 8, 88. 25610393 10.3389/fninf.2014.00088 PMC4285115

[hbm25879-bib-0034] Heed, T. , Buchholz, V. N. , Engel, A. K. , & Röder, B. (2015). Tactile remapping: From coordinate transformation to integration in sensorimotor processing. Trends in Cognitive Sciences, 19(5), 251–258. 25843541 10.1016/j.tics.2015.03.001

[hbm25879-bib-0035] Hu, C. , Di, X. , Eickhoff, S. B. , Zhang, M. , Peng, K. , Guo, H. , & Sui, J. (2016). Distinct and common aspects of physical and psychological self‐representation in the brain: A meta‐analysis of self‐bias in facial and self‐referential judgements. Neuroscience & Biobehavioral Reviews, 61, 197–207. 26695384 10.1016/j.neubiorev.2015.12.003

[hbm25879-bib-0036] Johnson‐Frey, S. H. , Maloof, F. R. , Newman‐Norlund, R. , Farrer, C. , Inati, S. , & Grafton, S. T. (2003). Actions or hand‐object interactions? Human inferior frontal cortex and action observation. Neuron, 39(6), 1053–1058. 12971903 10.1016/s0896-6273(03)00524-5

[hbm25879-bib-0037] Jokisch, D. , Daum, I. , & Troje, N. F. (2006). Self recognition versus recognition of others by biological motion: Viewpoint‐dependent effects. Perception, 35(7), 911–920. 16970200 10.1068/p5540

[hbm25879-bib-0038] Kalenzaga, S. , Sperduti, M. , Anssens, A. , Martinelli, P. , Devauchelle, A.‐D. , Gallarda, T. , … Krebs, M.‐O. (2015). Episodic memory and self‐reference via semantic autobiographical memory: Insights from an fMRI study in younger and older adults. Frontiers in Behavioral Neuroscience, 8, 449. 25628546 10.3389/fnbeh.2014.00449 PMC4292587

[hbm25879-bib-0039] Kanayama, N. , & Hiromitsu, K. (2021). Triadic body representations in the human cerebral cortex and peripheral nerves. In Y. Ataria , S. Tanaka , & S. Gallagher (Eds.), Body schema and body image: New directions (p. 133). Oxford University Press.

[hbm25879-bib-0040] Kriegeskorte, N. , Goebel, R. , & Bandettini, P. (2006). Information‐based functional brain mapping. Proceedings of the National Academy of Sciences, 103(10), 3863–3868. 10.1073/pnas.0600244103 PMC1383651 16537458

[hbm25879-bib-0041] Lee, S.‐H. , & Baker, C. I. (2016). Multi‐voxel decoding and the topography of maintained information during visual working memory. Frontiers in Systems Neuroscience, 10, 2. 26912997 10.3389/fnsys.2016.00002 PMC4753308

[hbm25879-bib-0042] Legrand, D. , & Ruby, P. (2009). What is self‐specific? Theoretical investigation and critical review of neuroimaging results. Psychological Review, 116(1), 252–282. 19159156 10.1037/a0014172

[hbm25879-bib-0043] Leshikar, E. D. , & Duarte, A. (2012). Medial prefrontal cortex supports source memory accuracy for self‐referenced items. Social Neuroscience, 7(2), 126–145. 21936739 10.1080/17470919.2011.585242 PMC3701388

[hbm25879-bib-0044] Limanowski, J. , & Blankenburg, F. (2013). Minimal self‐models and the free energy principle. Frontiers in Human Neuroscience, 7, 547. 10.3389/fnhum.2013.00547 PMC3770917 24062658

[hbm25879-bib-0045] Limanowski, J. , & Blankenburg, F. (2016). Integration of visual and proprioceptive limb position information in human posterior parietal, premotor, and extrastriate cortex. Journal of Neuroscience, 36(9), 2582–2589. 26937000 10.1523/JNEUROSCI.3987-15.2016 PMC6604875

[hbm25879-bib-0046] Lockwood, P. L. , Wittmann, M. K. , Apps, M. A. , Klein‐Flügge, M. C. , Crockett, M. J. , Humphreys, G. W. , & Rushworth, M. F. (2018). Neural mechanisms for learning self and other ownership. Nature Communications, 9(1), 1–11. 10.1038/s41467-018-07231-9 PMC6232114 30420714

[hbm25879-bib-0047] Lu, X. , Huang, J. , Yi, Y. , Shen, M. , Weng, X. , & Gao, Z. (2016). Holding biological motion in working memory: An fMRI study. Frontiers in Human Neuroscience, 10, 251. 27313520 10.3389/fnhum.2016.00251 PMC4887503

[hbm25879-bib-0048] Macey, P. M. , Macey, K. E. , Kumar, R. , & Harper, R. M. (2004). A method for removal of global effects from fMRI time series. NeuroImage, 22(1), 360–366. 15110027 10.1016/j.neuroimage.2003.12.042

[hbm25879-bib-0049] Macuga, K. L. , & Frey, S. H. (2011). Selective responses in right inferior frontal and supramarginal gyri differentiate between observed movements of oneself vs. another. Neuropsychologia, 49(5), 1202–1207. 21237185 10.1016/j.neuropsychologia.2011.01.005 PMC3078186

[hbm25879-bib-0050] Martinelli, P. , Sperduti, M. , & Piolino, P. (2013). Neural substrates of the self‐memory system: New insights from a meta‐analysis. Human Brain Mapping, 34(7), 1515–1529. 10.1002/hbm.22008 22359397 PMC6870171

[hbm25879-bib-0051] Mattan, B. , Quinn, K. A. , Apperly, I. A. , Sui, J. , & Rotshtein, P. (2015). Is it always me first? Effects of self‐tagging on third‐person perspective‐taking. Journal of Experimental Psychology. Learning, Memory, and Cognition, 41(4), 1100–1117. 10.1037/xlm0000078 25528086

[hbm25879-bib-0052] Molenberghs, P. , Cunnington, R. , & Mattingley, J. B. (2012). Brain regions with mirror properties: A meta‐analysis of 125 human fMRI studies. Neuroscience & Biobehavioral Reviews, 36(1), 341–349. 21782846 10.1016/j.neubiorev.2011.07.004

[hbm25879-bib-0053] Molnar‐Szakacs, I. , Iacoboni, M. , Koski, L. , & Mazziotta, J. C. (2005). Functional segregation within pars opercularis of the inferior frontal gyrus: Evidence from fMRI studies of imitation and action observation. Cerebral Cortex, 15(7), 986–994. 15513929 10.1093/cercor/bhh199

[hbm25879-bib-0054] Morita, T. , Asada, M. , & Naito, E. (2020). Right‐hemispheric dominance in self‐body recognition is altered in left‐handed individuals. Neuroscience, 425, 68–89. 31809726 10.1016/j.neuroscience.2019.10.056

[hbm25879-bib-0055] Myers, N. E. , Stokes, M. G. , & Nobre, A. C. (2017). Prioritizing information during working memory: Beyond sustained internal attention. Trends in Cognitive Sciences, 21(6), 449–461. 28454719 10.1016/j.tics.2017.03.010 PMC7220802

[hbm25879-bib-0056] Neisser, U. (1988). Five kinds of self‐knowledge. Philosophical Psychology, 1(1), 35–59.

[hbm25879-bib-0057] Northoff, G. , Heinzel, A. , De Greck, M. , Bermpohl, F. , Dobrowolny, H. , & Panksepp, J. (2006). Self‐referential processing in our brain—A meta‐analysis of imaging studies on the self. NeuroImage, 31(1), 440–457. 16466680 10.1016/j.neuroimage.2005.12.002

[hbm25879-bib-0058] Payne, S. , Tsakiris, M. , & Maister, L. (2017). Can the self become another? Investigating the effects of self‐association with a new facial identity. Quarterly Journal of Experimental Psychology, 70(6), 1085–1097. 10.1080/17470218.2015.1137329 26822152

[hbm25879-bib-0059] Pelphrey, K. A. , Morris, J. P. , Michelich, C. R. , Allison, T. , & McCarthy, G. (2005). Functional anatomy of biological motion perception in posterior temporal cortex: An fMRI study of eye, mouth and hand movements. Cerebral Cortex, 15(12), 1866–1876. 15746001 10.1093/cercor/bhi064

[hbm25879-bib-0060] Philippi, C. L. , Duff, M. C. , Denburg, N. L. , Tranel, D. , & Rudrauf, D. (2012). Medial PFC damage abolishes the self‐reference effect. Journal of Cognitive Neuroscience, 24(2), 475–481. 21942762 10.1162/jocn_a_00138 PMC3297026

[hbm25879-bib-0061] Pitcher, D. , & Ungerleider, L. G. (2020). Evidence for a third visual pathway specialized for social perception. Trends in Cognitive Sciences, 25(2), 100–110. 33334693 10.1016/j.tics.2020.11.006 PMC7811363

[hbm25879-bib-0062] Qin, P. , & Northoff, G. (2011). How is our self related to midline regions and the default‐mode network? NeuroImage, 57(3), 1221–1233. 21609772 10.1016/j.neuroimage.2011.05.028

[hbm25879-bib-0063] Qin, P. , Wang, M. , & Northoff, G. (2020). Linking bodily, environmental and mental states in the self: A three‐level model based on a meta‐analysis. Neuroscience & Biobehavioral Reviews, 115, 77–95. 10.1016/j.neubiorev.2020.05.004 32492474

[hbm25879-bib-0064] Quesque, F. , & Brass, M. (2019). The role of the temporoparietal junction in self‐other distinction. Brain Topography, 32(6), 943–955. 31676934 10.1007/s10548-019-00737-5

[hbm25879-bib-0065] Riggall, A. C. , & Postle, B. R. (2012). The relationship between working memory storage and elevated activity as measured with functional magnetic resonance imaging. Journal of Neuroscience, 32(38), 12990–12998. 22993416 10.1523/JNEUROSCI.1892-12.2012 PMC3470886

[hbm25879-bib-0066] Rizzolatti, G. , & Sinigaglia, C. (2010). The functional role of the parieto‐frontal mirror circuit: Interpretations and misinterpretations. Nature Reviews Neuroscience, 11(4), 264–274. 20216547 10.1038/nrn2805

[hbm25879-bib-0067] Saygin, A. P. (2007a). Brain areas involved in biological motion perception: What is involved and what is necessary. Journal of Vision, 7(9), 492.

[hbm25879-bib-0068] Saygin, A. P. (2007b). Superior temporal and premotor brain areas necessary for biological motion perception. Brain, 130(9), 2452–2461. 17660183 10.1093/brain/awm162

[hbm25879-bib-0069] Saygin, A. P. , Wilson, S. M. , Hagler, D. J. , Bates, E. , & Sereno, M. I. (2004). Point‐light biological motion perception activates human premotor cortex. Journal of Neuroscience, 24(27), 6181–6188. 15240810 10.1523/JNEUROSCI.0504-04.2004 PMC6729669

[hbm25879-bib-0070] Schäfer, S. , Wentura, D. , & Frings, C. (2017). Distinctiveness effects in self‐prioritization. Visual Cognition, 25(1–3), 399–411.

[hbm25879-bib-0071] Schmidt, T. T. , & Blankenburg, F. (2018). Brain regions that retain the spatial layout of tactile stimuli during working memory–A 'tactospatial sketchpad'? NeuroImage, 178, 531–539. 29860081 10.1016/j.neuroimage.2018.05.076

[hbm25879-bib-0072] Schmidt, T. T. , Schröder, P. , Reinhardt, P. , & Blankenburg, F. (2020). Rehearsal of tactile working memory: Premotor cortex recruits two dissociable neuronal content representations. Human Brain Mapping, 42(1), 245–258. 10.1002/hbm.25220 33009881 PMC7721226

[hbm25879-bib-0073] Schmidt, T. T. , Wu, Y.‐H. , & Blankenburg, F. (2017). Content‐specific codes of parametric vibrotactile working memory in humans. Journal of Neuroscience, 37, 1167–1117. 10.1523/JNEUROSCI.1167-17.2017 PMC6596612 28893928

[hbm25879-bib-0074] Schwoebel, J. , & Coslett, H. B. (2005). Evidence for multiple, distinct representations of the human body. Journal of Cognitive Neuroscience, 17(4), 543–553. 15829076 10.1162/0898929053467587

[hbm25879-bib-0075] Shi, Z. , Zhou, A. , Han, W. , & Liu, P. (2011). Effects of ownership expressed by the first‐person possessive pronoun. Consciousness and Cognition, 20(3), 951–955. 10.1016/j.concog.2010.12.008 21251853

[hbm25879-bib-0076] Shultz, S. , Lee, S. M. , Pelphrey, K. , & McCarthy, G. (2011). The posterior superior temporal sulcus is sensitive to the outcome of human and non‐human goal‐directed actions. Social Cognitive and Affective Neuroscience, 6(5), 602–611. 21097958 10.1093/scan/nsq087 PMC3190213

[hbm25879-bib-0077] Sokolov, A. A. , Zeidman, P. , Erb, M. , Ryvlin, P. , Friston, K. J. , & Pavlova, M. A. (2018). Structural and effective brain connectivity underlying biological motion detection. Proceedings of the National Academy of Sciences, 115(51), E12034–E12042. 10.1073/pnas.1812859115 PMC6305007 30514816

[hbm25879-bib-0078] Sugiura, M. , Sassa, Y. , Jeong, H. , Miura, N. , Akitsuki, Y. , Horie, K. , … Kawashima, R. (2006). Multiple brain networks for visual self‐recognition with different sensitivity for motion and body part. NeuroImage, 32(4), 1905–1917. 16806977 10.1016/j.neuroimage.2006.05.026

[hbm25879-bib-0079] Sui, J. , He, X. , & Humphreys, G. W. (2012). Perceptual effects of social salience: Evidence from self‐prioritization effects on perceptual matching. Journal of Experimental Psychology. Human Perception and Performance, 38(5), 1105–1117. 10.1037/a0029792 22963229

[hbm25879-bib-0080] Sui, J. , & Humphreys, G. W. (2015). The integrative self: How self‐reference integrates perception and memory. Trends in Cognitive Sciences, 19(12), 719–728. 10.1016/j.tics.2015.08.015 26447060

[hbm25879-bib-0081] Sui, J. , Liu, M. , Mevorach, C. , & Humphreys, G. W. (2015). The salient self: The left intraparietal sulcus responds to social as well as perceptual‐salience after self‐association. Cerebral Cortex, 25(4), 1060–1068. 24165832 10.1093/cercor/bht302

[hbm25879-bib-0082] Sui, J. , Rotshtein, P. , & Humphreys, G. W. (2013). Coupling social attention to the self forms a network for personal significance. Proceedings of the National Academy of Sciences of the United States of America, 110(19), 7607–7612. 10.1073/pnas.1221862110 23610386 PMC3651422

[hbm25879-bib-0083] Symons, C. S. , & Johnson, B. T. (1997). The self‐reference effect in memory: A meta‐analysis. Psychological Bulletin, 121(3), 371–394. 9136641 10.1037/0033-2909.121.3.371

[hbm25879-bib-0084] Tsakiris, M. (2010). My body in the brain: A neurocognitive model of body‐ownership. Neuropsychologia, 48(3), 703–712. 19819247 10.1016/j.neuropsychologia.2009.09.034

[hbm25879-bib-0085] Tsakiris, M. (2017). The multisensory basis of the self: From body to identity to others. The Quarterly Journal of Experimental Psychology, 70(4), 597–609. 27100132 10.1080/17470218.2016.1181768 PMC5214748

[hbm25879-bib-0086] Uluç, I. , Schmidt, T. T. , Wu, Y.‐H. , & Blankenburg, F. (2018). Content‐specific codes of parametric auditory working memory in humans. NeuroImage, 183, 254–262. 30107259 10.1016/j.neuroimage.2018.08.024

[hbm25879-bib-0087] Uluç, I. , Velenosi, L. A. , Schmidt, T. T. , & Blankenburg, F. (2020). Parametric representation of tactile numerosity in working memory. eNeuro, 7(1). ENEURO.0090‐19.2019. 10.1523/ENEURO.0090-19.2019 PMC7029184 31919053

[hbm25879-bib-0088] Vaina, L. M. , Solomon, J. , Chowdhury, S. , Sinha, P. , & Belliveau, J. W. (2001). Functional neuroanatomy of biological motion perception in humans. Proceedings of the National Academy of Sciences, 98(20), 11656–11661. 10.1073/pnas.191374198 PMC58785 11553776

[hbm25879-bib-0089] van Kemenade, B. M. , Muggleton, N. , Walsh, V. , & Saygin, A. P. (2012). Effects of TMS over premotor and superior temporal cortices on biological motion perception. Journal of Cognitive Neuroscience, 24(4), 896–904. 22264195 10.1162/jocn_a_00194

[hbm25879-bib-0090] Vanrie, J. , & Verfaillie, K. (2004). Perception of biological motion: A stimulus set of human point‐light actions. Behavior Research Methods, Instruments, & Computers, 36(4), 625–629. 10.3758/bf03206542 15641407

[hbm25879-bib-0091] Woźniak, M. (2019). How to grow a self: development of the self in a Bayesian brain. PsyArXiv. 1–48. 10.31234/osf.io/6e3ad

[hbm25879-bib-0092] Woźniak, M. , & Hohwy, J. (2020). Stranger to my face: top‐down and bottom‐up effects underlying prioritization of images of one's face. PLoS One, 15(7), e0235627. 10.1371/journal.pone.0235627 32645034 PMC7347180

[hbm25879-bib-0093] Woźniak, M. , & Knoblich, G. (2019). Self‐prioritization of fully unfamiliar stimuli. Quarterly Journal of Experimental Psychology, 72, 2110–2120. 10.1177/1747021819832981 30727822

[hbm25879-bib-0094] Woźniak, M. , & Knoblich, G. (2021). Self‐prioritization depends on assumed task‐relevance of self‐association. Psychological Research Psychologische Forschung. Advance online publication. 10.1007/s00426-021-01584-5 34491432

[hbm25879-bib-0095] Woźniak, M. , Kourtis, D. , & Knoblich, G. (2018). Prioritization of arbitrary faces associated to self: An EEG study. PLoS One, 13(1), e0190679. 29293670 10.1371/journal.pone.0190679 PMC5749812

[hbm25879-bib-0096] Wu, Y.‐H. , Uluç, I. , Schmidt, T. T. , Tertel, K. , Kirilina, E. , & Blankenburg, F. (2018). Overlapping frontoparietal networks for tactile and visual parametric working memory representations. NeuroImage, 166, 325–334. 29107771 10.1016/j.neuroimage.2017.10.059

[hbm25879-bib-0097] Yankouskaya, A. , Humphreys, G. , Stolte, M. , Stokes, M. , Moradi, Z. , & Sui, J. (2017). An anterior–posterior axis within the ventromedial prefrontal cortex separates self and reward. Social Cognitive and Affective Neuroscience, 12(12), 1859–1868. 29040796 10.1093/scan/nsx112 PMC5716107

[hbm25879-bib-0098] Yankouskaya, A. , & Sui, J. (2021). Self‐positivity or self‐negativity as a function of the medial prefrontal cortex. Brain Sciences, 11(2), 264. 33669682 10.3390/brainsci11020264 PMC7922957

[hbm25879-bib-0099] Yaoi, K. , Osaka, M. , & Osaka, N. (2015). Neural correlates of the self‐reference effect: Evidence from evaluation and recognition processes. Frontiers in Human Neuroscience, 9, 383. 26167149 10.3389/fnhum.2015.00383 PMC4481146

[hbm25879-bib-0100] Yin, S. , Bi, T. , Chen, A. , & Egner, T. (2021). Ventromedial prefrontal cortex drives the prioritization of self‐associated stimuli in working memory. Journal of Neuroscience, 41(9), 2012–2023. 33462089 10.1523/JNEUROSCI.1783-20.2020 PMC7939096

[hbm25879-bib-0101] Zhao, S. , Uono, S. , Li, C. , Yoshimura, S. , & Toichi, M. (2018). The influence of self‐referential processing on attentional orienting in frontoparietal networks. Frontiers in Human Neuroscience, 12, 199. 29867422 10.3389/fnhum.2018.00199 PMC5962753

[hbm25879-bib-0102] Zhou, A. , Shi, Z. , Zhang, P. , Liu, P. , Han, W. , Wu, H. , … Xia, R. (2010). An ERP study on the effect of self‐relevant possessive pronoun. Neuroscience Letters, 480(2), 162–166. 10.1016/j.neulet.2010.06.033 20561562

